# Identifying Gaps in Bolivian Neurosurgery: A Systematic Review and Proposed Framework for Resource Implementation

**DOI:** 10.7759/cureus.93947

**Published:** 2025-10-06

**Authors:** Caleigh S Roach, Jacob J Shawwa, Connor S Nee, Jacqueline L Brenner, Vagif Kazimli, Shreyas Chetan, Jorge D Brun, Joyce Koueik, Richard Moser, Bermans Iskandar, Jorge D Brun, Victor M Lu

**Affiliations:** 1 Department of Neurosurgery, University of Miami Miller School of Medicine, Miami, USA; 2 Miller School of Medicine, University of Miami, Miami, USA; 3 Department of Interventional Radiology, University of Miami Miller School of Medicine, Miami, USA; 4 Department of Neurological Surgery, Hospital del Niño “Dr. Ovidio Aliaga Uria”, La Paz, BOL; 5 Department of Neurosurgery, University of Wisconsin, Madison, USA; 6 Department of Neurosurgery, University of Massachusetts, Worcester, USA; 7 Department of Neurological Surgery, University of Wisconsin School of Medicine and Public Health, Madison, USA

**Keywords:** bolivia, global neurosurgery, health equity, latin america, neurosurgery, technology

## Abstract

Neurosurgical systems in low- and middle-income countries (LMICs) often face challenges in infrastructure, technology, workforce, and continuity of care. Bolivia, an LMIC in South America, remains underrepresented in global neurosurgical research. Correspondingly, this study aimed to systematically review the neurosurgical literature of Bolivia to identify existing strengths, key gaps, and opportunities for targeted investment and technological implementation. A systematic review was conducted using PubMed, Scopus, and Embase to identify studies reporting original data on neurosurgical care in Bolivia. Data were extracted across the following domains: study design, patient population, surgical volume, institutional capacity, technology access, outcomes, and reported barriers or recommendations. Descriptive statistics and thematic synthesis were used to characterize trends and identify priority areas. Eleven studies met the inclusion criteria, with a heavy research focus on pediatric neurosurgery (98% of study population). Public institutions represented 47% of centers, and 56% of services were urban based. Standard procedures included trauma and hydrocephalus management. Major system gaps included a lack of advanced surgical technology (neuronavigation absent in 46%, microsurgery in 36%), limited imaging access (64%), and high loss to follow-up (21%). Reported barriers spanned infrastructure, workforce, infection control, and delays in referral. Recommendations emphasized imaging expansion, surgical training, telemedicine, and protocol standardization. Neurosurgical care in Bolivia is characterized by concentrated pediatric research, variable infrastructure, and fragmented systems. This review identifies key opportunities for capacity-building and targeted collaboration to strengthen neurosurgical care in Bolivia and similar LMICs.

## Introduction and background

Equitable access to neurosurgical care remains a critical yet unmet dimension of global health, particularly in low- and middle-income countries (LMICs) where limitations in infrastructure, workforce, and diagnostics disproportionately affect surgical outcomes [1-2]. Within Latin America, Bolivia maintains one of the lowest neurosurgical workforce densities, estimated at just 0.4 neurosurgeons per 100,000 population, well below the internationally recommended threshold of 0.7 per 100,000 [3-4]. Despite regional progress in academic exchange and workforce development, Bolivia remains underrepresented in global surgical discourse and receives minimal international investment in neurosurgical training or infrastructure [5-6]. In contrast to neighboring countries with established neuroendoscopy and spine programs, for example, Bolivian neurosurgical practice continues to face foundational deficiencies in surgical instrumentation and postoperative continuity of care [6-9].

Historical accounts reflect an uneven trajectory of Latin American neurosurgical development, where specialized services remain concentrated in more developed countries and are frequently reliant on short-term mission-based models [10-11]. Bolivia remains notably absent from regional surgical capacity-building initiatives and is sparsely represented in the literature, indicating deficiencies in both clinical resources and scholarly attention [1,3]. While select institutional series and pediatric-focused reports from Bolivia have been published, no prior effort has comprehensively synthesized the scope of neurosurgical care delivery across geographic, institutional, and clinical dimensions.

To address this, the present study provides the first systematic synthesis of neurosurgical literature from Bolivia. By outlining institutional capacity, procedural volume, workforce engagement, and reported outcomes, this review establishes a foundational framework for policy development, technology implementation, and sustainable academic partnerships within the broader context of global neurosurgical progress [12-14].

## Review

Materials and methods

Search Strategy and Eligibility Criteria

**Figure 1:**
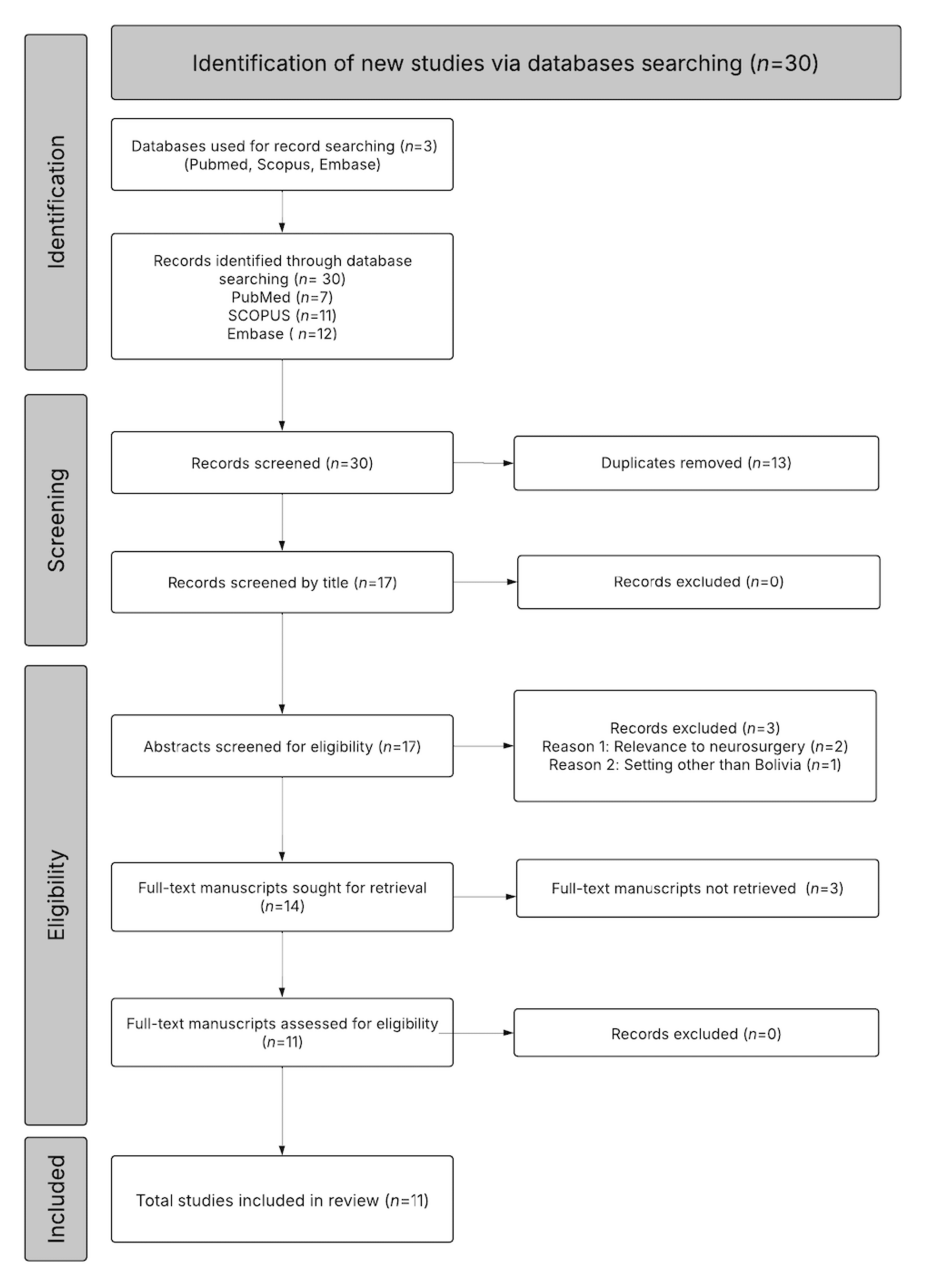
PRISMA 2020 flow diagram for systematic review of neurosurgical gaps in Bolivia. Flowchart showing the study selection process for the systematic review, including database search, screening, eligibility assessment, and final inclusion. PRISMA: Preferred Reporting Items for Systematic Reviews and Meta-Analyses.

This systematic review was conducted under Preferred Reporting Items for Systematic Reviews and Meta-Analyses (PRISMA) guidelines to identify all available literature on neurosurgical care in Bolivia. A comprehensive search was performed across PubMed/MEDLINE, Embase, and Scopus from inception through January 2025. A diagram of the PRISMA flow strategy can be found in Figure 1, and the complete search strategy is available in Table 1. Eligible studies included those reporting original data on neurosurgical clinical care, systems infrastructure, training, or surgical outcomes within Bolivia. Commentaries, editorials, and studies that lack country-specific relevance were excluded. Although only a few Bolivian medical journals exist, none are indexed in major international databases, and the country does not have dedicated neurosurgical journals. As a result, locally produced work is seldom visible internationally, which contributes to the limited amount of indexed literature. Therefore, gray literature (including conference proceedings, institutional reports, and non-indexed regional journals) was manually reviewed to enhance the capture of regionally relevant material, and no language-specific restrictions were imposed. One Spanish-language study was included and translated by a bilingual reviewer; no additional translation support was required. Since this study relied solely on previously published data, it did not require ethical review or institutional board approval. Meta-analysis was not performed owing to the substantial heterogeneity in study designs, reporting standards, and small sample sizes across the 11 included studies. For these same reasons, formal preregistration in a traditional systematic review registry such as PROSPERO was not pursued, as the project did not align with conventional meta-analytic frameworks.

**Table 1:** Search strategies used for systematic review across three databases. This table outlines the specific Boolean search strings used in PubMed, Embase, and Scopus to identify relevant literature on neurosurgical care in Bolivia.

Database	Search Strategy
PubMed	(Bolivia[Title/Abstract]) AND (neurosurgery[Title/Abstract] OR neurosurgical[Title/Abstract] OR "neurosurgical care"[Title/Abstract] OR "neurosurgical services"[Title/Abstract] OR "neurosurgical procedures"[Title/Abstract])
Embase	('bolivia') AND ('neurosurgery' OR 'neurosurgical' OR 'neurosurgical care' OR 'neurosurgical services' OR 'neurosurgical procedures')
Scopus	TITLE-ABS-KEY(bolivia) AND TITLE-ABS-KEY(neurosurgery OR neurosurgical OR "neurosurgical care" OR "neurosurgical services" OR "neurosurgical procedures")

Study Selection and Data Extraction

Two reviewers independently screened titles and abstracts. Full texts of potentially eligible studies were reviewed for final inclusion, with discrepancies resolved through a consensus and a third reviewer. Inclusion and exclusion criteria are detailed in Table 2. A standardized data extraction matrix collected publication characteristics (year, study design, setting), institutional attributes (ownership, geographic location), patient demographics, procedural volumes, diagnostic categories, perioperative outcomes, follow-up metrics, and reported resource availability. Particular attention was paid to author affiliation and authorship order (first/senior) to assess the nationality of academic participation. Resource variables (e.g., imaging, intensive care unit (ICU) access, operative technologies) were only coded when explicitly reported as present or absent; studies that did not address a specific resource were excluded from that denominator to avoid misclassification.

**Table 2:** Eligibility criteria for inclusion in the systematic review. This table outlines the predefined inclusion and exclusion criteria used to guide study selection.

	Inclusion Criteria	Exclusion Criteria
Outcome measure	Articles specifically focused on neurosurgery or neurosurgical care services in Bolivia	Studies without a specific focus on Bolivia
Factors of interest	Surgical care access, delivery, or workforce; neurosurgical procedures or outcomes; system-level or hospital-level analysis of neurosurgical care; and pre-hospital, referral, and post-operative neurosurgical pathways	Studies unrelated to neurosurgery; general trauma or spine studies, unless directly involving neurosurgical care
Population	Healthcare systems, hospitals, or clinics in Bolivia; and national and regional healthcare initiatives within Bolivia related to neurosurgery	Studies not directly discussing neurosurgery in Bolivia; and veterinary or non-human neurosurgical studies
Setting	Bolivia as the primary setting or focus	Studies focused on healthcare settings outside Bolivia without including Bolivia as a case study
Study design	Empirical studies; reviews focusing on neurosurgical implementation barriers; and case reports/series and reviews	Editorials, commentaries, or opinion pieces with no empirical data; case studies focusing on non-healthcare industries or non-implementation contexts
Type of publication	Peer-reviewed journal articles, and full-text studies	Abstracts without full texts available; non-peer-reviewed articles

Data Analysis

Quantitative synthesis involved descriptive statistics and pooled frequencies to summarize study characteristics, clinical activity, outcomes, and system gaps. Outcome measures were averaged across studies to account for reporting variability and heterogeneity in data quality. A separate thematic analysis was conducted to identify recurring barriers and proposed interventions across studies. Extracted qualitative content was coded inductively and organized into higher-order domains informed by system-level frameworks in global neurosurgery. Proposed recommendations were similarly categorized, with attention to contextual relevance and implementation feasibility. Recurrent patterns were compared across studies to inform a targeted framework for future intervention in Bolivian neurosurgical systems.

Risk of Bias and Quality Assessment

Formal risk of bias assessment was applied using the Risk of Bias in Non-randomized Studies of Interventions (ROBINS-I) tool [15] to each of the included studies. Two reviewers independently assessed all seven domains (confounding, selection of participants, classification of interventions, deviations from intended interventions, missing data, outcome measurement, and selection of the reported result), with disagreements resolved by consensus. Ratings were coded as low, moderate, or serious risk of bias. Furthermore, due to deficiency of available research, overlapping authorship across several studies was present. This was documented and acknowledged due to the potential for institutional bias in reporting.

Results

Study Characteristics

A comprehensive literature search yielded 30 unique studies, of which 11 met inclusion criteria following full-text review. These studies [16-26], published between 1979 and 2024, constitute the currently available literature on neurosurgical care in Bolivia. Data were extracted from each study and collated as depicted in Table 3. Four of the included studies were case series [16,20-21,25], four were cohort studies [16,20,22-23], and only two studies were cross-sectional analysis by design [17-18]. Four of 11 included studies were multisite [17-18,20,24], and only one study took place during a short-term surgical trip [19]. Bolivian academic participation was variable: Bolivian authors served as first authors in five studies [21-24,26], and senior authors in seven studies [16,20-24,26], reflecting growing national engagement yet persistent dependence on international collaboration.

**Table 3:** Extracted data from neurosurgical studies on Bolivia from 1979 to 2024 (N = 11). This table summarizes key characteristics from 11 neurosurgical studies in Bolivia, including study design, author and center affiliations, geographic distribution, surgical details, patient demographics, diagnoses, and outcomes. OR: operating room.

Variable Category	Variable	% (n)
Study design	Cohort	36.4 (4)
	Cross-sectional	18.2 (2)
	Case series	36.4 (4)
	Multisite study	36.4 (4)
	Multicenter study	27.3 (3)
	Short-term surgical trip	9.1 (1)
Author affiliation from Bolivia	First author	45.5 (5)
	Senior author	63.6 (7)
	Other author	63.6 (7)
Institutional affiliation of neurosurgical centers	Public	47.4 (9)
	Private	10.5 (2)
	University	26.3 (5)
	Charity	5.3 (1)
	Unknown	10.5 (2)
Geographic service classification	Urban	56.3 (9)
	Rural	12.5 (2)
	Both	25 (4)
	Unknown	6.3 (1)
Regional (department-level) location of neurosurgical centers	La Paz	38.5 (5)
	Cochabamba	15.4 (2)
	Santa Cruz	38.5 (5)
	Tarija	7.7 (1)
Institutional affiliation of operating neurosurgeons	Home institution	69.4 (86)
	External institution	30.6 (38)
Distribution of neurosurgical procedures by type	Trauma procedures	32.5 (256)
	Elective procedures	13.1 (103)
	Other or unknown	54.4 (429)
Distribution of age groups represented in study populations	Pediatric	97.8 (716)
	Adult	2.2 (16)
Primary neurosurgical diagnoses reported	Traumatic brain injury/head trauma	24.0 (168)
	Hydrocephalus	39.0 (274)
	Neoplastic intracranial mass	12.4 (94)
	Spine trauma	15.6 (11)
	Elective spine	9.2 (65)
	Cerebral abscess	0.8 (6)
	Peripheral nerve	11.4 (80)
	Spine infection	0.7 (5)
Documented patient demographics	Male patients	55.9 (409)
	Female patients	44.1 (323)
Patient outcomes and clinical courses reported	Intraoperative complications	1.7%
	Shunt failure	12.0%
	Postoperative complications (non-infectious)	39.1%
	Infection	20.2%
	Hospital stay complications	28.3%
	Return to OR	21.8%
	Overall survival	72.3%
	Mortality	28.0%
	Neurosurgical mortality	3.7%
	Average length of stay (days)	21.0%
	Follow-up	54.0%
	Lost to follow-up	20.8%

Institutional Capacity and Geographic Distribution

**Figure 2:**
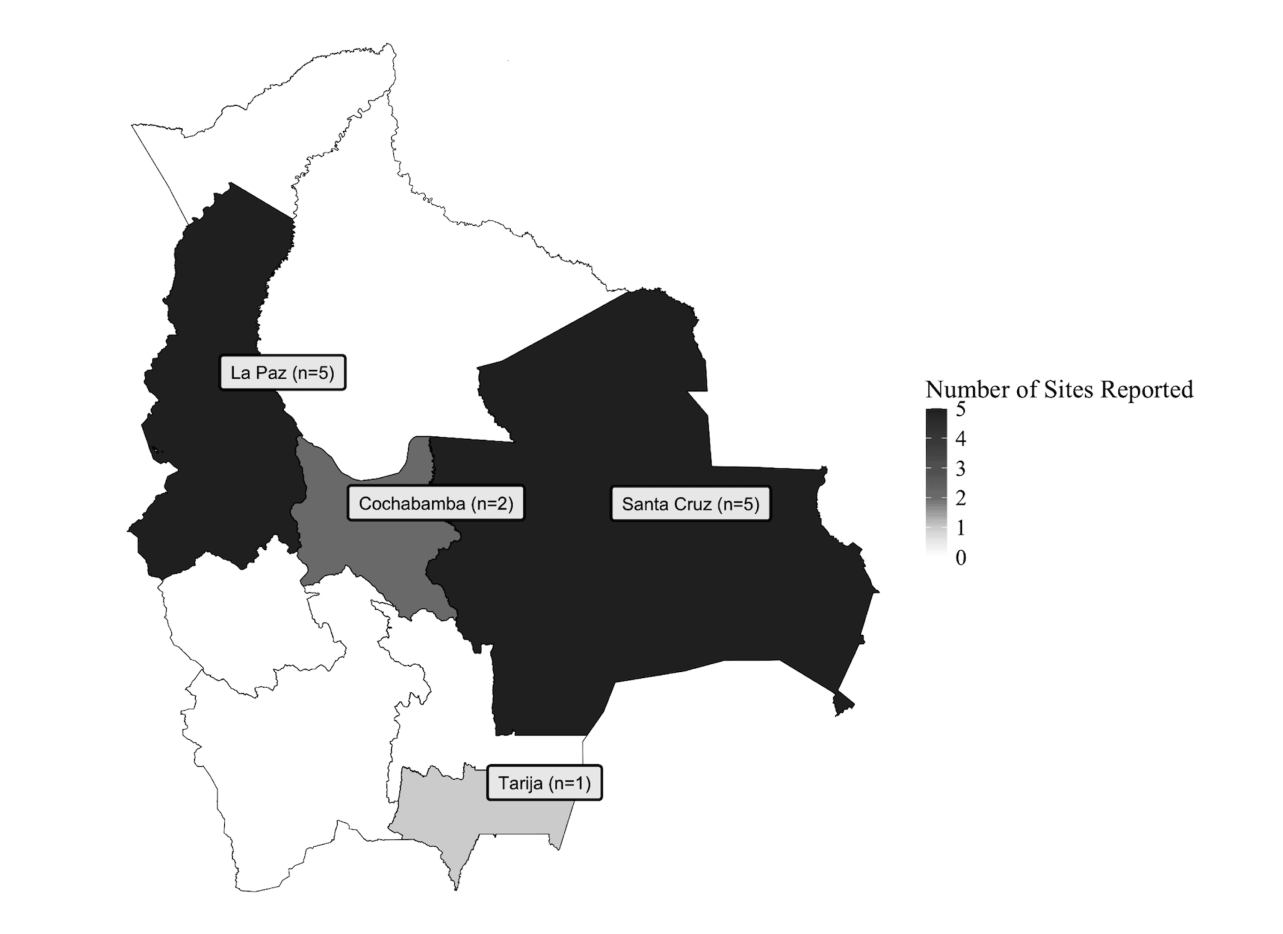
Regional distribution of hospitals involved in neurosurgery studies in Bolivia. Choropleth map of Bolivia indicating the geographic locations of hospitals featured in the included neurosurgical studies, illustrating regional representation across departments.

Most neurosurgical institutions represented were public (47%), with fewer affiliated with universities (26%), private systems (11%), or charities (5%). Services were concentrated in urban areas (56%), with limited rural representation (13%), reinforcing known geographic disparities in Bolivian healthcare delivery. Departmental distribution revealed that neurosurgical activity was primarily confined to La Paz and Santa Cruz (39% each), with minimal representation from other departments such as Tarija (8%) and Cochabamba (15%), as seen in Figure 2. Regarding operating personnel, 69% of procedures were performed by surgeons affiliated with the hosting institution, while 31% involved external neurosurgeons in the context of academic partnerships or mission-based care.

Procedural Volume and Diagnostic Distribution

Across all included studies, trauma-related interventions accounted for 33% of cases, with elective procedures comprising just 13%. Over half (54%) were classified as “other” or “unknown,” underscoring limitations in procedural granularity and documentation. Diagnostically, hydrocephalus was the most treated condition (39%), followed by traumatic brain injury (24%), intracranial neoplasms (12%), spine trauma (16%), and peripheral nerve lesions (11%). Notably, across all published studies, pediatric patients constituted 98% of all cases, with only 2% of procedures performed on adults, reflecting a skewed focus on congenital and developmental conditions.

Clinical Outcomes and Postoperative Course

Clinical outcomes were reported inconsistently, but when available, highlighted significant perioperative morbidity. Postoperative complications (non-infectious) were documented in 39% of cases, and infection rates reached 20%. Shunt failure occurred in 12% of hydrocephalus procedures. Reoperation rates were high at 22%, while complications during hospitalization (e.g., wound breakdown, pressure ulcers) occurred in 28% of patients. Neurosurgical mortality was reported at 4% (range 0%-8%), with an overall survival rate of 72% (range 38%-90%). Continuity of care was a major challenge: only 54% of patients had any documented follow-up, and 21% were explicitly lost to follow-up, significantly limiting outcome interpretation and quality assurance. The average length of stay was noted in only a subset of cases, reported at 21 days.

Infrastructure and Technological Capacity

Substantial variation in institutional resources was observed across studies, as shown in Table 4. CT imaging was available in 80% of studies reporting diagnostic capacity, while MRI access was significantly more limited (20%). Intraoperative imaging and advanced surgical technology were inconsistently available: ultrasound, endoscopy, and neuronavigation were each reported in 25% or fewer of applicable settings. Endoscopic equipment was present in 50% of operative centers, but no studies reported access to microsurgical instrumentation. Operative environments were largely low-tech, and digital integration was minimal: 75% of institutions used paper records exclusively, and only one institution was noted to use an electronic medical record system presently. Ventilator-supported ICU care was universally available in the subset of studies that discussed ICU settings, although only 75% of institutions were explicitly described as having infection control protocols. Notably, telehealth capabilities were absent from all included reports.

**Table 4:** Reported institutional capacity and available resources across included studies (N = 11). This table outlines the availability of imaging, operative, ICU, and record-keeping resources across neurosurgical centers described in the 11 included studies, highlighting infrastructure relevant to diagnostics, surgical capabilities, postoperative care, and health information systems. CT: computed tomography; MRI: magnetic resonance imaging; U/S: ultrasound; ICU: intensive care unit; EMR: electronic medical records; EHR: electronic health records.

Category	Resource	Reported Present, % (n)	Reported Absent, % (n)
Imaging	CT	80.0% (4)	20.0% (1)
	MRI	20.0% (1)	80.0% (4)
	U/S	50.0% (1)	50.0% (1)
Operative	Intraoperative MRI	16.7% (1)	83.3% (5)
	Intraoperative CT	25.0% (1)	75.0% (3)
	Intraoperative U/S	50.0% (1)	50.0% (1)
	Microsurgery	0.0% (0)	100% (3)
	Endoscopy	50.0% (3)	50.0% (3)
	Neuronavigation	25.0% (1)	75.0% (3)
ICU	Ventilation	100% (4)	0.0% (0)
	Infection control protocol	75.0% (3)	25.0% (1)
Record keeping	Paper records	75.0% (3)	25.0% (1)
	EMR/EHR	25.0% (1)	75.0% (3)
	Telehealth	0.0% (0)	100% (6)

Identified Neurosurgical Gaps

**Figure 3:**
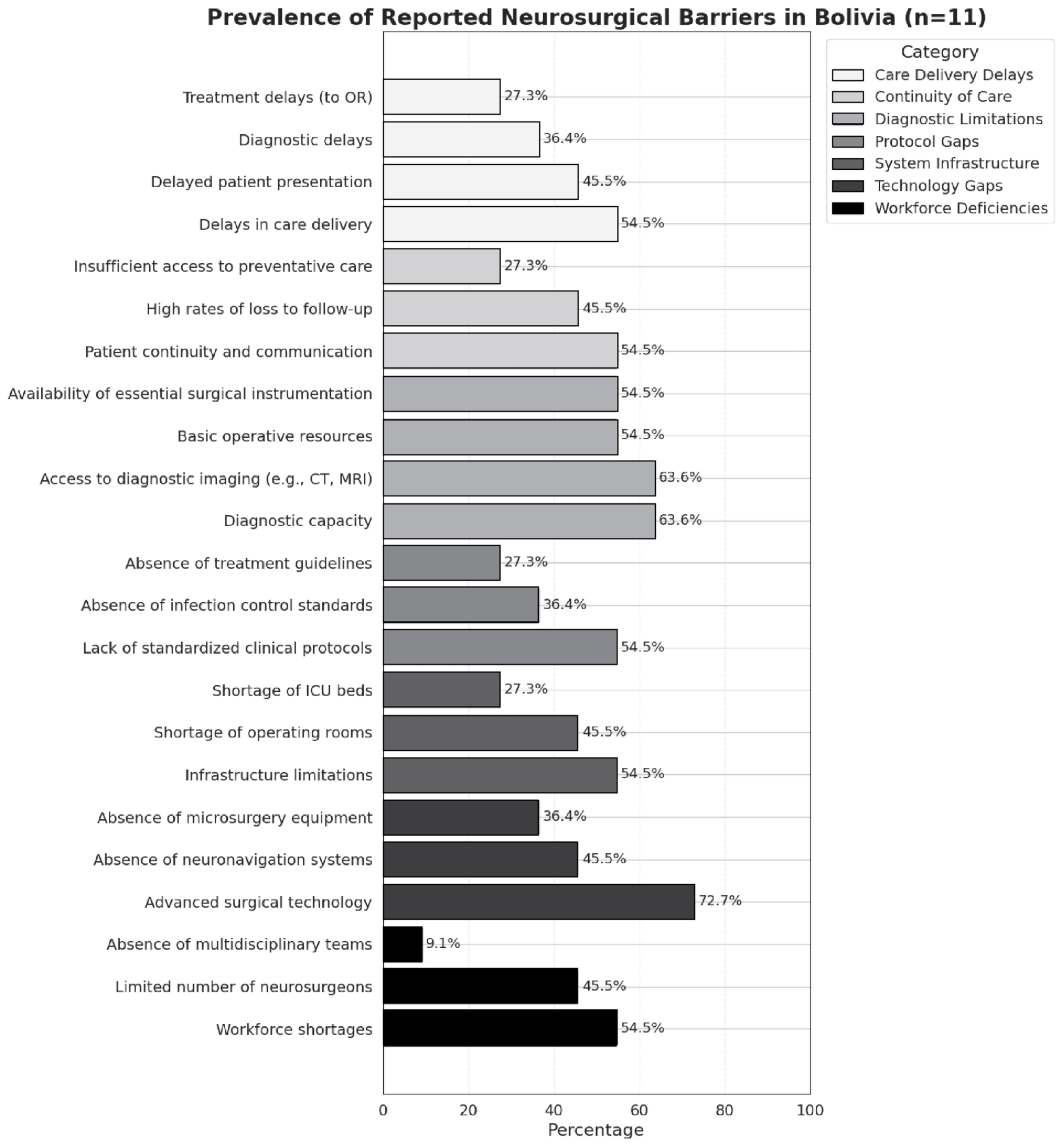
Cross-study prevalence of reported neurosurgical barriers in Bolivia. Composite figure summarizing thematic barriers to neurosurgical care identified across studies, categorized into infrastructure, technology, diagnostics, care delivery, workforce, continuity of care, and clinical protocols. OR: operating room; CT: computed tomography; MRI: magnetic resonance imaging; ICU: intensive care unit.

Analysis of reported system gaps revealed recurrent deficiencies across several core domains, which are comprehensively summarized in Table 5 and visually represented in Figure 3. Infrastructure limitations were prominent, with 46% of studies reporting a shortage of operating rooms. ICU limitations were identified in 27% of studies, and diagnostic imaging access was inadequate in 64% of studies, with CT or MRI often unavailable or delayed. Regarding surgical equipment, 55% reported that basic surgical instrumentation was unavailable or insufficient. Advanced technologies such as microsurgical tools and neuronavigation systems were absent in 36% and 46% of studies, respectively. Delays in care delivery were frequently cited; 46% of studies mentioned prolonged time to initial presentation, 36% described delays in diagnostic imaging, and 27% reported operative delays. Workforce shortages were notable, with 46% of studies indicating a limited number of neurosurgeons and 9% describing a lack of multidisciplinary support. Continuity of care was another frequent concern, with 45% of studies highlighting high rates of loss to follow-up and 27% pointing to insufficient access to preventative care. Additionally, 36% reported an absence of infection control standards, and 27% noted a lack of formal clinical protocols, pointing to critical gaps in perioperative safety systems.

**Table 5:** Reported neurosurgical gaps in included studies (N = 11). This table summarizes the most reported neurosurgical system gaps across the 11 included studies, including infrastructure deficits, limited diagnostic and operative capacity, delayed care delivery, workforce shortages, continuity of care challenges, and lack of standardized clinical protocols. ICU: intensive care unit; CT: computed tomography; MRI: magnetic resonance imaging.

Neurosurgical Gap	Sub-category Gap	Studies, % (n)
Infrastructure		54.5% (6)
	Shortage of operating rooms	45.5% (5)
	Shortage of ICU beds	27.3% (3)
Diagnostic capacity		63.6% (7)
	Access to diagnostic imaging (e.g., CT, MRI)	63.6% (7)
Basic operative resources		54.5% (6)
	Availability of essential surgical instrumentation	54.5% (6)
Advanced surgical technology		72.7% (8)
	Absence of neuronavigation systems	45.5% (5)
	Absence of microsurgery equipment	36.4% (4)
Time to care delivery		54.5% (6)
	Delayed patient presentation (prolonged time to initial presentation)	45.5% (5)
	Diagnostic delays (prolonged time to initial imaging)	36.4% (4)
	Treatment delays (prolonged time to operative intervention)	27.3% (3)
Workforce		54.5% (6)
	Limited number of neurosurgeons	45.5% (5)
	Absence of multidisciplinary teams	9.1% (1)
Patient continuity and communication		54.5% (6)
	High rates of loss to follow-up	45.5% (5)
	Insufficient access to preventative care	27.3% (3)
Standardized clinical protocols		54.5% (6)
	Absence of treatment guidelines	27.3% (3)
	Absence of infection control standards	36.4% (4)

Thematic Barriers to Care

Thematic synthesis of reported neurosurgical barriers revealed six core categories describing recurring structural and systemic challenges. Infrastructure and resource deficits were the most prevalent, reported in 82% of studies and included operating room scarcity, limited ICU bed availability, and unreliable access to diagnostic tools such as CT and MRI. Fragmentation in follow-up and continuity of care was noted in 73% of studies, often manifesting as poor longitudinal tracking, high loss to follow-up, and limited systems for postoperative surveillance. Workforce shortages, limited training opportunities, and governance gaps were identified in 64% of studies; these were frequently compounded by a lack of team-based care models and inadequate access to continuing education or subspecialty support. Protocol and infection control deficiencies were described in 55% of studies, with some institutions lacking basic perioperative standards. Socioeconomic barriers, cited in 64% of studies, encompassed affordability, transportation, and indirect costs and were often exacerbated by rural isolation and geographic inaccessibility. Referral system fragmentation and poor coordination between facilities were identified in 46% of studies, while 36% referenced population-specific access limitations, including disparities at altitude and among indigenous populations. Together, the prominent barriers describe a multifactorial landscape of neurosurgical inequity. Detailed incidence of specific neurosurgical barriers and categorized direct quotes from each study are provided in Tables 6, 7.

**Table 6:** Incidence of specific neurosurgical barriers identified across all included studies. This table lists individual barriers to neurosurgical care in Bolivia as reported in the 11 studies included in the systematic review, along with the number of times each was mentioned. CT: computed tomography; MRI: magnetic resonance imaging; EMS: emergency medical services; PPE: personal protective equipment.

Barrier	Number of mentions (n)
Nonfunctional or unavailable CT scanner	10
General imaging limitations	4
Loss to follow-up or poor follow-up infrastructure	4
Lack of perioperative or infection control protocols	4
High postoperative infection rates	3
Lack of MRI	2
Absence of telemedicine	2
Barriers to shunt care	2
Resident burnout	2
Limited access to radiation therapy	2
Limited access to chemotherapy	1
Lack of EMS or prehospital care	1
Reliance on paper records/imaging	1
Centralization of neurosurgical care	1
Low preventive care	1
Provider income reduction	1
Mistreatment or abuse of residents	1
Lack of institutional PPE	1
Lack of endoscopic tools	1
Lack of neuronavigation	1
Lack of ultrasound (portable or intraoperative)	1
Lack of postgraduate programs	1

**Table 7:** Categorized neurosurgical barrier quotes extracted from each study. Categorized direct quotes from the included studies describing neurosurgical barriers. MRI: magnetic resonance imaging; CT: computed tomography; NTD: neglected tropical diseases; PPE: personal protective equipment; post-op: postoperative; ICU: intensive care unit.

Study	Barrier A	Barrier B	Barrier C
1 [16]	Lack of MRI and nonfunctional CT scanner.	No telemedicine infrastructure available.	Few residents trained uniformly; lack of postgraduate structure.
2 [17]	Low follow-up rates due to geographic and telemedicine limitations.	Lack of perioperative protocols and sterile technique variability.	Centralization of care limits rural access to neurosurgery.
3 [18]	High postoperative infection rates due to protocol variability.	Patients must travel and pay for radiation/chemo post-op.	No preventive folic acid use; high NTD burden.
4 [19]	Resident burnout and mental health issues are prevalent.	No system for abuse reporting or psychological support.	Neurosurgical societies lack authority in regulating specialty.
5 [20]	Widespread mistreatment and lack of reporting systems.	Surgical education is inconsistent; bootcamps unsustainable.	High cost of electrical stimulation; not introduced.
6 [21]	Elective surgeries halted due to pandemic.	Lack of post-op protocols led to high infection rates.	Burnout among residents led to poor mental health outcomes.
7 [22]	No institutional PPE protocols; providers buy their own.	No access to neuronavigation or modern operative tools.	High-altitude surgical risks and endemic infections.
8 [23]	Most neurosurgeons lost income during pandemic.	Approval delays for needed surgical supplies.	Neurosurgical beds and ICU resources are insufficient.
9 [24]	Poor access to tertiary care and imaging due to cost.	Radiation therapy not available at hospital; high cost externally.	No modern imaging system for postoperative follow-up.
10 [25]	Only shunt treatment available due to lack of endoscope.	Lack of follow-up hinders shunt complication monitoring.	Rural patients can’t afford or reach follow-up care.
11 [26]	CT scanner often unavailable; imaging only in hardcopy.	No portable ultrasound or MRI; only paper imaging records.	Limited access to adjuvant therapy impacts survival.

Formal Risk of Bias Assessment

**Figure 4:**
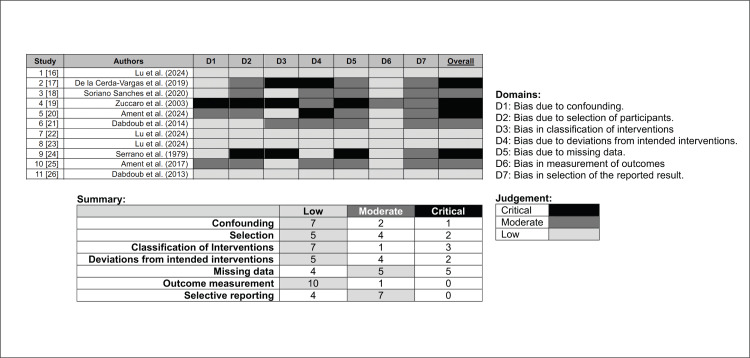
ROBINS-I risk of bias assessment across included studies (N = 11). Traffic-light plot of risk-of-bias judgments using the ROBINS-I tool. Each row represents one included study; each column represents one ROBINS-I domain: D1 = Confounding, D2 = Selection of participants, D3 = Classification of interventions, D4 = Deviations from intended interventions, D5 = Missing data, D6 = Outcome measurement, D7 = Selection of reported results. Green indicates low risk of bias, yellow indicates moderate risk, and red indicates serious risk. Overall study judgments reflect the highest risk rating across domains. ROBINS-I: Risk of Bias in Non-randomized Studies of Interventions.

The ROBINS-I assessments revealed significant insights into the quality of available studies, indicating that only four out of 11 exhibited a consistently low risk of bias across all domains. The majority of studies were classified as having a moderate risk, primarily due to issues such as inadequate outcome measurement, inadequate control for confounding variables, and ambiguities in intervention classification. Furthermore, four studies were marked with a critical risk, stemming from challenges in various areas, including publication year and insufficient rigor in both intervention and outcome assessment. These findings are summarized in Figure 4.

Discussion

This systematic review represents the first comprehensive synthesis of neurosurgical literature in Bolivia, aiming to identify and characterize national-level gaps in infrastructure, technology, clinical protocols, and workforce. The impetus for this study arose from the lack of consolidated data regarding neurosurgical capacity in the country, which hinders both policy development and international collaboration. By systematically capturing published and gray literature, this review provides a foundational appraisal of Bolivia’s neurosurgical landscape, offering insight into critical deficiencies and generating a platform for targeted, context-specific health system strengthening.

A key observation was the large focus of reported patients' outcomes being that of pediatric neurosurgery, with nearly 98% of reported procedures involving children. Numerically, this was driven by the large dataset recently created by Lu et al. who was able to provide multiple cohort studies to the literature on different pediatric topics such as hydrocephalus, myelomeningocele, and medulloblastoma [16,21-23]. While these conditions are clinically significant, their overrepresentation likely misrepresents the broader neurosurgical burden within Bolivia. This is because the other included studies found in this review did not provide the same size or granularity in other neurosurgical subspecialties which one would expect to see in other more mature national neurosurgical literature. This large-study bias should be considered then, and signal that the Bolivian neurosurgical literature remains in a nascent stage limiting inferences based solely on patient outcome data right now.

Equally concerning was the systemic underdevelopment and inconsistency of neurosurgical infrastructure. Fewer than one-third of studies reported access to advanced tools such as intraoperative neuronavigation or imaging modalities like CT or MRI. Paper-based recordkeeping and a lack of integrated digital health systems were commonly described [16,23,26], along with a notable absence of standardized infection control or ICU protocols. These findings mirror earlier stages of neurosurgical development in LMICs such as Uganda, which has since improved outcomes through national investment in education, diagnostic imaging, and registry-based documentation [12]. The Bolivian context remains fragmented and underdeveloped, lacking coordinated infrastructure development or technology-sharing frameworks.

Continuity of care and long-term patient outcomes emerged as critical blind spots in the current literature. Despite the significant burden of postoperative complications (particularly infections and reoperations), fewer than half of the studies included structured follow-up, and from these, 21% of patients were explicitly lost to follow-up [16,20,22-23]. This mirrors findings in other LMIC neurosurgical settings, where transportation barriers, financial limitations, and system fragmentation undermine longitudinal care [13-14]. Bolivia’s mountainous geography and rural-urban access divide likely exacerbate these challenges. While some countries have piloted mobile health (mHealth) or regional follow-up hubs to improve patient retention [18], no such initiatives were identified within the Bolivian literature. Moreover, the sporadic use of electronic medical records limits opportunities for audit, benchmarking, and registry-based research, all of which are increasingly emphasized in global neurosurgery [7,8,20].

The findings of this review offer multiple implications for health policy and clinical practice. Expanding access to imaging modalities, particularly CT and MRI, is foundational to both emergency and elective neurosurgical care. Standardizing perioperative documentation, infection control protocols, and follow-up procedures could significantly enhance patient safety and data reliability. Investments in formal postgraduate training programs and neurosurgical education (especially through long-term partnerships with high-income country (HIC) institutions) may alleviate critical workforce shortages. Furthermore, incorporating telemedicine and mobile health platforms into postoperative care could help address the rural-urban access divide and improve continuity of care, a persistent challenge in Bolivia’s mountainous and variable geography.

Proposed Recommendations for Bolivian Neurosurgery

Despite substantial challenges, many studies proposed actionable and context-specific recommendations to improve neurosurgical care in Bolivia. Expanding diagnostic capacity, particularly access to CT, MRI, and intraoperative imaging, was emphasized in 18% of studies, with additional calls to incorporate endoscopy into routine surgical practice. Training and international collaboration were recommended in 27% of studies, which advocated for the creation of domestic neurosurgical residency programs, formal postgraduate pathways, and academic partnerships through long-term twinning models. Financial and policy reforms were endorsed in 36% of studies, highlighting the need for increased investment in workforce retention, surgical equipment procurement, and economic security for neurosurgical trainees and providers. Telemedicine and structured follow-up programs were recommended in 27% of studies as strategies to address geographic isolation and continuity-of-care breakdowns. Evidence from Uganda shows that telehealth and electronic medical records (EMR) implementation only succeeded when paired with structured, peer-led training programs and continuous professional development, which improved provider confidence, reduced technology hesitancy, and supported long-term system sustainability [27]. Adapting similar education-focused models could ensure that Bolivian telemedicine initiatives are not only launched but sustained. Improvements in clinical documentation, infection control standards, and perioperative protocol development were also recurring themes, supporting broader efforts to strengthen research infrastructure and standardize care delivery. Notably, 55% of studies offered uncategorized but highly relevant context-specific recommendations, including the engagement of local non-governmental organizations (NGOs), the development of altitude-adapted neurosurgical protocols, government-led prevention strategies, and expanded access to adjuvant therapies such as chemotherapy and radiotherapy for neurosurgical oncology patients. Further specific recommendations and categorized quotes extracted from the studies are detailed in Tables 8, 9. These suggestions reflect a growing awareness within the Bolivian neurosurgical community of both the complexity of existing barriers and the need for sustainable, locally driven solutions.

**Table 8:** Frequency of specific neurosurgical recommendations proposed across all included studies. This table compiles individual recommendations offered by the 11 studies included in the systematic review, highlighting the breadth of proposed solutions to improve neurosurgical care in Bolivia. CT: computed tomography; MRI: magnetic resonance imaging; HIC: high-income country; NTD: neglected tropical diseases; PPE: personal protective equipment; NGO: non-governmental organization.

Recommendation	Number of Mentions (n)
Upgrade imaging (CT, MRI, endoscope)	1
Improve collaborative decision-making using tech	1
Promote access to journals/books for young neurosurgeons	1
Annual course expansion and institute formation	1
Form international donation-based equipment networks	1
Establish surgical education centers and low-cost sets	1
Create formal postgraduate training systems	1
Train Bolivian surgeons in HIC centers	1
Advanced neurophysiology research for altitude care	1
Further investigation into neurosurgery at high altitudes	1
Improve follow-up infrastructure and systems	1
Improve follow-up via telehealth	1
Introduce endoscopic equipment for hydrocephalus	1
Improve imaging modality access post-op	1
Expand access to imaging for shunt monitoring	1
Patient and family education for adjuvant therapy	1
Form multidisciplinary tumor boards	1
Invest in telemedicine	1
Twinning programs with HIC hospitals	1
Multi-national study collaboration	1
Environmental monitoring (soil/fertilizers)	1
Government-supported NTD prevention programs	1
Neurosurgery leadership in care improvement	1
Conduct prospective cost-effectiveness research	1
Improve surgical training and hygiene/infection prophylaxis	1
Develop physician economic support mechanisms	1
Ensure PPE availability and protocols	1
Implement telemedicine programs	1
Acknowledge burnout as a global health issue	1
Take action against abuse/discrimination	1
Establish protection/support mechanisms for residents	1
Develop infection control protocols	1
Leverage local NGOs for bootcamp coordination	1

**Table 9:** Categorized neurosurgical recommendations extracted from each included study. Categorized direct quotes from the included studies describing neurosurgical recommendations. CT: computed tomography; MRI: magnetic resonance imaging; PPE: personal protective equipment; NTD: neglected tropical diseases; HIC: high-income country; NGO: non-governmental organization.

Study	Recommendation A	Recommendation B	Recommendation C
1 [16]	Upgrade imaging (CT, MRI, endoscope)	Invest in telemedicine	Develop infection control protocols
2 [17]	Establish protection/support mechanisms for residents	Take action against abuse/discrimination	Acknowledge burnout as a global health issue
3 [18]	Implement telemedicine programs	Ensure PPE availability and protocols	Develop physician economic support mechanisms
4 [19]	Improve surgical training and hygiene/infection prophylaxis	Conduct prospective cost-effectiveness research	Neurosurgery leadership in care improvement
5 [20]	Government-supported NTD prevention programs	Environmental monitoring (soil/fertilizers)	Multi-national study collaboration
6 [21]	Twinning programs with HIC hospitals	Form multidisciplinary tumor boards	Improve collaborative decision-making using tech
7 [22]	Patient and family education for adjuvant therapy	Expand access to imaging for shunt monitoring	Improve imaging modality access post-op
8 [23]	Introduce endoscopic equipment for hydrocephalus	Improve follow-up via telehealth	Improve follow-up infrastructure and systems
9 [24]	Further investigation into neurosurgery at high altitudes	Advanced neurophysiology research for altitude care	Train Bolivian surgeons in HIC centers
10 [25]	Create formal postgraduate training systems	Establish surgical education centers and low-cost sets	Form international donation-based equipment networks
11 [26]	Annual course expansion and institute formation	Promote access to journals/books for young neurosurgeons	Leverage local NGOs for bootcamp coordination

Limitations

However, several limitations must be acknowledged. The small number of studies limits generalizability and increases susceptibility to publication bias. The pediatric dominance in reported cases reflects the recent published pediatric series rather than true national disease distribution, restricting broader conclusions about Bolivia’s neurosurgical epidemiology. Variability in outcome reporting precluded formal meta-analysis and limited the comparability of study findings. Language and access barriers may have excluded additional local studies, particularly those not digitized. To minimize this, we did not restrict by language and included one Spanish-language study; however, the absence of a multilingual or centralized Bolivian database limits the ability to ensure full inclusion of all relevant literature. Lastly, recurring authorship across several studies, along with the potential overrepresentation of internationally co-authored articles, may have led to institutional bias, selection bias, and limitations in the diversity of perspectives represented. Despite these limitations, this review provides the first structured appraisal of Bolivia's neurosurgical capacity and research priorities, offering a baseline for future study and intervention.

## Conclusions

This systematic review reveals that the neurosurgical landscape in Bolivia is marked by a scarcity of published research, infrastructural inconsistency, and fragmented clinical pathways. While there are encouraging signs of growing national authorship and isolated institutional strength, critical gaps persist across surgical equipment and technology domains, diagnostics, follow-up systems, and workforce development and training. The findings underscore the need for coordinated, locally anchored strategies to expand access to technology, develop a national neurosurgical registry, and foster sustainable training programs. Addressing these deficits through targeted investment and international collaboration with multicenter research and partnerships will be crucial for advancing neurosurgical equity in Bolivia and similar LMICs. This study provides a foundation upon which future research, policy design, and cross-sector partnerships can be built to strengthen the future of neurosurgery in the region.
